# Facile Synthesis of Green Fluorescent Carbon Dots and Their Application to Fe^3+^ Detection in Aqueous Solutions

**DOI:** 10.3390/nano12091487

**Published:** 2022-04-27

**Authors:** Shuai Ye, Mingming Zhang, Jiaqing Guo, Jun Song, Pengju Zeng, Junle Qu, Yue Chen, Hao Li

**Affiliations:** 1 Center for Biomedical Optics and Photonics (CBOP) & College of Physics and Optoelectronic Engineering, Key Lab of Optoelectronics Devices and Systems of Ministry of Education/Guangdong Province, Shenzhen University, Shenzhen 518060, China; yes121@szu.edu.cn (S.Y.); zhang130731@tom.com (M.Z.); gjq@szu.edu.cn (J.G.); songjun@szu.edu.cn (J.S.); zengpj@szu.edu.cn (P.Z.); jlqu@szu.edu.cn (J.Q.); 2 National Research Nuclear University MEPhI (Moscow Engineering Physics Institute), 115409 Moscow, Russia

**Keywords:** carbon dot, Fe^3+^, fluorescence spectrum, fluorescent probe, ions detection

## Abstract

Carbon dots (CDs), a class of fluorescent nanomaterials, have attracted widespread attention from researchers. Because of their unique chemical properties, these high-quality fluorescent probes are widely used for ion and molecule detection. Excess intake of many ions or molecules can cause harm to the human body. Although iron (in the form of Fe^3+^ ions) is essential for the human body, excess iron in the human body can cause many diseases, such as iron poisoning. In this study, we have synthesized fluorine and nitrogen co-doped carbon dots (FNCDs) by a hydrothermal method. These FNCDs exhibited good stability, selectivity, and anti-interference ability for Fe^3+^. Fe^3+^ could be detected in the range of 0.2–300 μM, and their detection limit is up to 0.08 μM. In addition, the recovery and relative standard deviation measured by the standard addition recovery method were not higher than 107.5% and 1.1%, respectively, indicating that FNCDs have good recovery and accuracy for Fe^3+^ detection.

## 1. Introduction

Carbon dots (CDs) are a new class of fluorescent nanomaterials that have widespread applications in fields such as photoelectric device fabrication [1,2], photodynamic therapy [3], living cell imaging [4,5,6], and biosensing [7,8,9], owing to their unique optical and chemical properties. In contrast to most luminescent materials, such as semiconductor quantum dots [10], precious metal nanoclusters, fluorescent proteins, and rare earth ions, CDs have unique optical properties, such as wavelength tunability in the visible region, two-photon emission, excellent photobleaching resistance, good photostability, and ability to exhibit photothermal effect. Most CDs are synthesized using low-cost carbon-based materials that contain abundant functional groups. Consequently, the surface of CDs has abundant functional groups, enabling them to effectively recognize ions and molecules and target specific organelles for imaging. Thus, CDs are being widely explored in the field of bioimaging and biosensing applications.

Fe is a common element in daily life and is an essential trace element in the human body. An average adult has approximately 4 to 5 mg of Fe, which is present in hemoglobin and actin. These two proteins are mainly used for oxygen transport, storage, and release. Therefore, Fe plays a crucial role in stimulating human metabolism; however, excess Fe in the human body can lead to serious diseases, such as Parkinson’s disease, cancer, and cirrhosis [11,12,13,14,15]. Because Fe is recycled efficiently, only trace amounts of Fe are excreted from the small intestine, rendering self-regulation difficult. Therefore, the quantitative detection of Fe is of great biological significance. For example, Ge et al. [16] used fresh tea leaves and urea as carbon and nitrogen sources, respectively, for the hydrothermal synthesis of CDs for Fe^3+^ detection. The detection range and detection limit of these CDs were 0–300 μM and 0.079 μM, respectively. Although a carbon dot has excellent detection range and detection limit, it is only used for the detection of Fe^3+^ in cells, and there is no detection data in actual water samples. Chen et al. [17] reported a method for the rapid synthesis of nitrogen-doped CDs by microwave-assisted pyrolysis using glutamic acid and ethylenediamine as carbon sources and N dopants, respectively. The Fe^3+^ detection range and detection limit of these CDs were 8–80 μM and 3.8 μM, respectively. It has been applied in intracellular and actual water samples. Although there are many application data of the carbon dots, their detection range and detection limit still have room for improvement. Zulfajri et al. [18] synthesized fluorescent CDs using cranberry beans, where the detection range and detection limit reached 30–600 μM and 9.55 μM, respectively. It also was applied to Fe^3+^ detection in actual water samples. Although the detection range is excellent, the detection limit was still high. Le Minh Tu Phan have developed nitrogen-doped CDs by using citric acid as a carbon source and polyethyleneimine molecular weight 1800 (PEI1800) as a nitrogen source [19]. The linear detection range and detection limit of the CDs for Fe^2+^ reached 0–50 μM and 0.702 μM. The Fe^2+^ detection in cells was also studied. Although only a few CDs can detect Fe^2+^, the detection range and detection limit of this carbon point need to be improved. In addition, this experiment also lacks the research results on the detection of Fe^2+^ in actual water samples. Therefore, the synthesis of a fluorescent CDs, which can detect Fe^3+^ in actual water samples and has a better detection range and detection limit, has important research significance.

In this work, we used 4,5-difluoro-o-phenylenediamine and ethylenediamine as precursors to synthesize green fluorescent fluorine and nitrogen co-doped carbon dots (FNCDs). FNCDs showed excellent detection ability for Fe^3+^. Compared with similar carbon dots that can detect Fe^3+^ in actual water samples, FNCDs have a better detection range or detection limit. In addition, the fitting degree of the linear relationship between the fluorescence intensity of FNCDs and Fe^3+^ concentration is very good. Therefore, the FNCDs have the advantages of a fast response, high accuracy, and high detection range for the detection of Fe^3+^.

## 2. Experimental Section

### 2.1. Materials

All reagents used in this study were of analytical grade and used in accordance with the standards. Metal salts (AgNO_3_, NaCl, KI, MnCl_2_·4H_2_O, CaCl_2_, MgCl_2_·6H_2_O, (CH_3_COO)_2_Ba, (CH_3_COO)_2_Pb, (CH_3_COO)_2_Sr, ZnCl_2_, CsBr, FeCl_3_·6H_2_O, NaOH, Na_2_HPO_4_, NaNO_2_, NaHCO_3_, LiCl, and NaF) and HCl were purchased from Macklin (Shanghai, China). 4,5-Difluoro-1,2 phenylenediamine was supplied by Macklin (Shanghai, China). Ethylenediamine was purchased from XIYA Reagent Co., Ltd., (Shandong, China).

### 2.2. Instruments

Transmission electron microscopy (TEM, FEI TECNAI G2 F20, Hillsboro, OR, USA), X-ray photoelectron spectroscopy (XPS, Thermo Fisher ESCALAB 250Xi, Waltham, MA, USA), Fourier-transform infrared (FT-IR) spectroscopy (Nicolet 5700 spectrometer, Waltham, MA, USA, mode: ATR, scans times: 64, resolution: 4 cm^−1^), ultraviolet-visible (UV-Vis) spectroscopy (UV-2550 Shimadzu, Osaka, Japan), fluorescence spectroscopy (PL, Varian Cary Eclipse Agilent, Santa Clara, CA, USA), and fluorescence lifetime (FL) measurements (FLS1000 photoluminescence spectrometer) were performed to determine the morphology, chemical composition, chemical structure, and optical properties of the samples. A laser-scanning confocal fluorescence microscope (Nikon A1R MP+, Tokyo, Japan) was used for cell imaging.

### 2.3. Synthesis of FNCDs

To synthesize FNCDs, 0.1 g of 4,5-difluoro-1,2 phenylenediamine and 0.1 g of ethylenediamine were added to a polytetrafluoroethylene-lined autoclave containing 15 mL of water, and the reaction was allowed to proceed at 180 °C for 12 h. After the reaction, the reaction kettle was naturally cooled to room temperature. The solution was centrifuged at 8000 rpm for 5 min, and the supernatant was collected. The centrifugation was repeated twice as above to remove large particles. The supernatant was dialyzed for three days against deionized water using a 500 Da membrane, and the deionized water was changed every 12 h during the dialysis. Finally, the dialysate was filtered through a 0.22 μM polyethersulfone aqueous membrane and lyophilized to obtain the FNCD powder.

### 2.4. Quantitative Detection of Fe^3+^

A constant concentration of FNCDs and different known concentrations of Fe^3+^ were mixed. The emission intensity at 512 nm, upon excitation at 418 nm, was measured. The detection limit of FNCDs for Fe^3+^ was calculated using the detection limit formula proposed by the International Union of Pure and Applied Chemistry [20]:

L = 3σ/k

Here, k represents the slope of the fluorescence intensity vs. Fe^3+^ concentration plot, and σ represents the standard deviation of the background sample. The standard deviation represents the deviation in the fluorescence intensity of the background sample at 512 nm for 20 measurements.

### 2.5. Detection of Fe^3+^ in Different Water Samples

Laboratory water samples and dormitory water samples were used as solvents to prepare Fe^3+^ solutions of different concentrations. A fixed concentration of FNCDs was added to the water samples and the reaction was allowed to proceed for 2 min, following which the fluorescence intensity at 512 nm was measured. The fluorescence intensity was substituted into the fitting equation obtained from the fluorescence intensity vs. Fe^3+^ concentration plot to obtain the ion concentration in the samples. Each experiment was performed in more than three groups to obtain the standard deviation and recovery rate.

## 3. Results and Discussion

### 3.1. Characterization of FNCDs

**Scheme 1:**
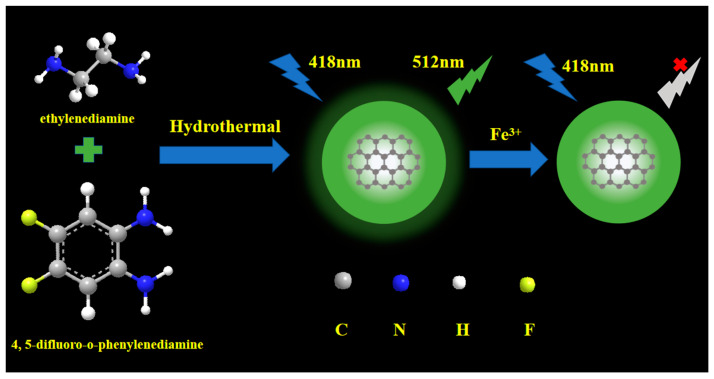
One-pot hydrothermal synthesis of green fluorescent FNCDs.

**Figure 1:**
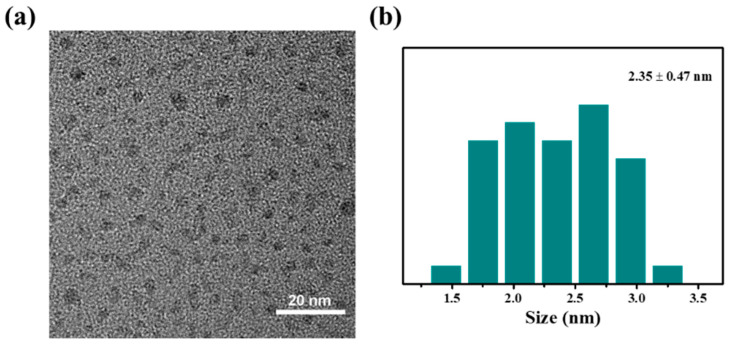
(**a**) TEM image and (**b**) particle size distribution histogram of FNCDs.

4,5-difluoro-1,2 phenylenediamine and ethylenediamine form carbon dots through high-temperature condensation and carbonization (Scheme 1). The morphology and particle size distribution of the FNCDs were characterized using TEM. The TEM image (Figure 1a) and particle size distribution (Figure 1b) revealed that the FNCDs had a relatively uniform particle size distribution (2.35 ± 0.47 nm) and good dispersion.

**Figure 2:**
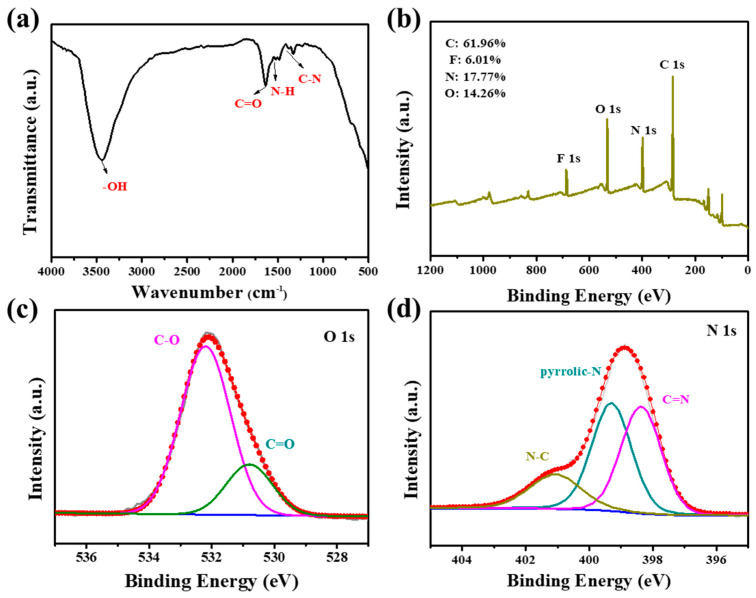
(**a**) FT-IR spectra and (**b**) XPS survey spectra of FNCDs. Corresponding high-resolution (**c**) O 1s and (**d**) N 1s XPS profiles.

The structure of the FNCDs was further characterized by FT-IR spectroscopy and XPS. The FT-IR spectrum showed a characteristic absorption peak of OH at 3450 cm^−1^. The peak at 1630 cm^−1^ can be attributed to the asymmetric C=O stretching vibration. The peaks at 1410 cm^−1^ correspond to the C–N stretching vibration. XPS was performed to analyze the chemical bonds and elemental composition. The C, O, N, and F contents in the synthesized FNCDs were 61.96%, 14.26%, 17.77%, and 6.01%, respectively (Figure 2b). The high-resolution C 1s XPS profile showed four peaks corresponding to C–C/C=C (284.7 eV), C–O/C–N (285.5 eV), C=O (286.7 eV), and C–F (288.6 eV), respectively (Figure S1a). Figure 2c shows the high-resolution O 1s spectra; the three peaks in this spectrum correspond to C=O (530.8 eV) and C–O (532.2 eV). The high-resolution N 1s spectrum showed peaks corresponding to C=N (398.3 eV), pyrrolic–N (399.3 eV), and N–C (401.1 eV). The peaks in the high-resolution F 1s spectrum can be ascribed to covalent C–F (687.1 eV) and semi-ionic C–F (685.0 eV) [21,22,23,24]. The FT-IR and XPS analyses confirmed that N and F were doped into the CDs and that many kinds of functional groups were produced.

### 3.2. Optical Characterization

**Figure 3:**
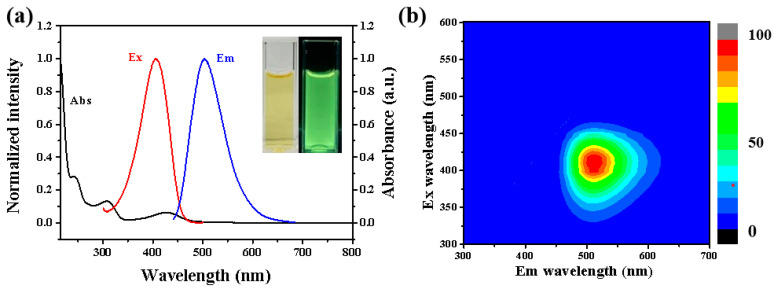
(**a**) Absorption (black), excitation (red), and emission (blue) spectra of the FNCDs. Inset: images of the N-CDs solution under visible light (**left**) and 365 nm UV light (**right**). (**b**) 3D fluorescence spectrum of the FNCDs (the colors from blue to red represent the fluorescence intensity from weak to strong).

The optical properties of the FNCDs were also studied. As evident from Figure 3a, the FNCDs exhibit clear excitation and emission peaks at 418 and 512 nm, respectively. The 3D spectrum suggests that the FNCDs were excitation-independent CDs (Figure 3b). The absorption spectrum showed three peaks at 245, 313, and 420 nm, corresponding to the π-π* transition, n-π* transition, and bandgap absorption, respectively. By comparing the absorption peak and excitation peak, it can be seen that the luminescence mechanism of the CDs is obviously different from that of semiconductor quantum dots or organic small molecules. This is because the luminescence mechanism of the CDs is determined by both nuclear and surface states.

We next attempted to determine if the synthesized FNCDs were suitable for the detection of Fe^3+^ in environmental samples. The change in the fluorescence intensity of the FNCDs in solutions with different NaCl concentrations (Figure S2a) suggests that the FNCDs have strong salt resistance. The change in the fluorescence intensity of FNCDs upon irradiation at 365 nm for different durations was examined (Figure S2b). Although the fluorescence intensity gradually decreases upon irradiation for more than 20 min, the time required for an emission scan does not exceed 1 min. Therefore, the FNCDs are suitable for the detection of Fe^3+^ in environmental samples. The change in the fluorescence intensity of FNCDs at different pH levels suggests that these CDs are highly stable in the pH range of 2–11 (Figure S2c). Fortunately, the pH of most of the solutions tested was in the neutral range. However, for the quantitative detection of Fe^3+^, it is also necessary to measure the fluorescence intensity of FNCDs in Fe^3+^ solution at different reaction times (Figure S2d). The fluorescence intensity of FNCDs remained constant till ~5 min of reaction with Fe^3+^, suggesting that the FNCDs have good reaction sensitivity and stability. Thus, FNCDs have excellent stability in different environments, rendering them suitable for ion or molecule detection.

### 3.3. Selectivity of Fe^3+^

**Figure 4:**
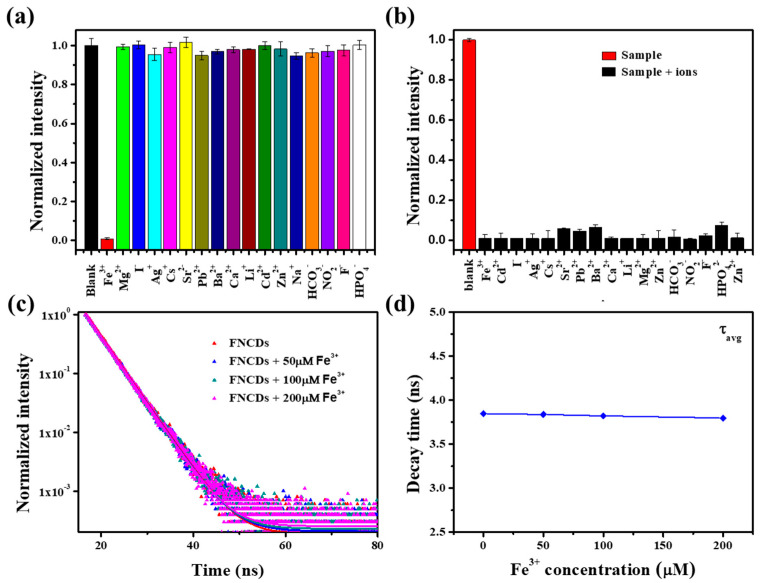
(**a**) Fluorescence intensity of FNCDs in the presence of different ions. (**b**) Change in the fluorescence intensity after adding different ions to the FNCDs/Fe^3+^ reaction system. (**c**) Fluorescence lifetime spectra of FNCDs and FNCDs with different concentrations Fe^3+^ (excitation and emission wavelengths: 450 and 512 nm). (**d**) The fluorescence lifetime of FNCDs after adding different concentrations of Fe^3+^.

Figure 4a shows the changes in the fluorescence intensity of the FNCDs in the presence of different ions (1 mM). The fluorescence intensity decreased drastically in the presence of Fe^3+^, whereas it remained almost same as that of the blank sample in the presence of other ions. This suggested the high selectivity of the FNCDs toward Fe^3+^ ions. The addition of different ions into the FNCDs/Fe^3+^ reaction system did not change the fluorescence intensity significantly (Figure 4b), indicating the good anti-interference ability of FNCDs.

**Figure 5:**
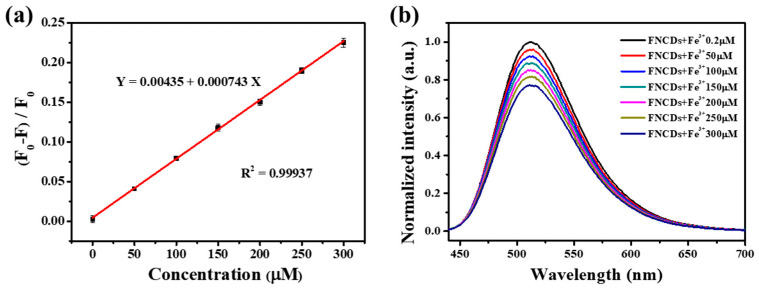
(**a**) Linear fitting of the relative fluorescence vs. concentration plot and (**b**) emission spectra of the FNCDs at various Fe^3+^ concentrations (from top to bottom: 0.2, 50, 100, 150, 200, 250, 300 μM).

Common fluorescence quenching mechanisms include inner filter effect (IFE), fluorescence resonance energy transfer (FRET), and photoinduced electron transfer (PET). To investigate the fluorescence quenching mechanism of FNCDs, the absorption spectrum and fluorescence lifetime of Fe^3+^ and FNCDs were measured. FRET and PET change with the fluorescence lifetime. However, when the excitation and emission wavelength are 450 nm and 512 nm respectively, the fluorescence lifetimes of FNCDs at different Fe^3+^ concentrations (0–200 μM) remained almost unchanged (Figure 4c,d). So, the quenching mechanism is not FRET and PET. In addition, the absorption spectra of FNCDs and Fe^3+^ overlap in the 300–408 nm range (Figure S3). Although the degree of overlap is not as good as that at short wavelengths (270–310 nm), the higher excitation light energy of the FNCDs will be absorbed with an increase in the Fe^3+^ concentration. The trend in the lifetime and the absorption spectra indicate static fluorescence quenching by the inner filter effect [25]. Consequently, the relationship between the fluorescence intensity of FNCDs and the concentration of Fe^3+^ was studied (Figure 5). Figure 5b shows that the fluorescence intensity of the FNCDs decreased linearly with increasing Fe^3+^ concentration. The plot could be fitted to the equation

Y=0.00435+0.000743 X

where X represents the concentration of Fe^3+^ and Y = (F_0_ − F)/F_0_. F_0_ represents the fluorescence intensity at [Fe^3+^] = 0, and F represents the fluorescence intensity at different Fe^3+^ concentrations. The correlation coefficient (R^2^) of the equation was 0.99937, suggesting a strong linear relationship between the fluorescence intensity and Fe^3+^ concentration in the range of 0.2–300 μM (Figure 5a). The detection limit and detection range were 0.08 μM and 0.2–300 μM, respectively. This detection range is wider than those of the previously reported fluorescent CDs for Fe^3+^ detection (Table S1).

Our results suggest that the synthesized FNCDs are a high-quality probe for Fe^3+^ detection. To determine the practical applicability of the FNCDs for Fe^3+^ detection in environmental samples, the Fe^3+^ concentration in laboratory water samples and dormitory water samples was analyzed by the standard addition recovery method. The results are presented in Table 1. Briefly, 40, 80, and 120 μM Fe^3+^ solutions were added to the laboratory water samples and dormitory water samples. The standard addition recoveries ranged from 95.8 to 97.7% and 93.6 to 107.5%, and the relative standard deviations were 0.6–1.0% and 0.3–1.1%, respectively. The recovery from the spiked samples was not higher than 107.5%, and the relative standard deviation was not higher than 1.1%. The good recovery and accuracy of FNCDs suggest their broad application prospect for Fe^3+^ detection in different water samples.

## 4. Conclusions

In general, green fluorescent FNCDs were synthesized by a hydrothermal method using 4, 5-difluoro-o-phenylenediamine and ethylenediamine. Owing to their excellent photostability, salt resistance, pH stability, Fe^3+^ selectivity, and anti-interference ability, FNCDs can be used to quantitatively detect Fe^3+^ in different water samples. The detection limit and detection range were 0.08 μM and 0.2–300 μM, respectively. When FNCDs were used to detect Fe^3+^ in dormitory water and laboratory water samples, the recovery and relative standard deviation were not higher than 107.5% and 1.1%, respectively. Compared with other carbon dots for Fe^3+^ detection in actual water samples, FNCDs have a better detection range or detection limit. The detection results in actual water samples show that FNCDs have excellent anti-interference ability. FNCDs also has broad application prospects for Fe^3+^ detection of other water samples. However, the detection limit of FNCDs is still relatively high, which limits the detection of FNCDs given trace Fe^3+^. If FNCDs can detect the Fe^3+^ of a cell, FNCDs are expected to have important applications in the research of Fe^3+^ diseases.

## Figures and Tables

**Table 1:** Recovery of Fe^3+^ in actual water samples.

Samples	Detection Amount (μM)	Adding Fe^3+^ (μM)	Found Fe^3+^ (μM)	Recovery (%)	RSD (*n* = 3, %)
Laboratory water	-	40	39.08	97.7	1.0
		80	77.85	97.3	0.7
		120	114.96	95.8	0.6
Dormitory water	-	40	37.43	93.6	0.3
		80	86.00	107.5	0.6
		120	125.69	104.7	1.1

## Data Availability

The data presented in this study are available on request from the corresponding author.

## Supplementary Materials

The following supporting information can be downloaded at: https://www.mdpi.com/article/10.3390/nano12091487/s1, Figure S1: High-resolution (a) C 1s and (b) F 1s of XPS spectra; Figure S2: FNCDs fluorescence intensity at (a) different NaCl concentration, (b) different light time, (c) dif-ferent pH and (d) different reaction time at 1mM Fe^3+^ concentration; Figure S3: Absorption spectra of Fe^3+^ (blue line), NFCDs (red line), and NFCDs quenched by Fe^3+^ (black line); Table S1: Fe^3+^ detection ability of different fluorescent CDs. References [26,27,28,29,30] are cited in the Supplementary Materials.
