# Supplementary material for: Facile Synthesis of Green Fluorescent Carbon Dots and Their Application to Fe3+ Detection in Aqueous Solutions
Source: Nanomaterials (Basel). 2022 Apr 27;12(9):1487. doi: 10.3390/nano12091487 (PMC9104042; doi:10.3390/nano12091487)
Supplement: Supplementary file 1 [file nanomaterials-12-01487-s001.zip › nanomaterials-1688853-supplementary.pdf]

nanomaterials

Article

Facile Synthesis of Green Fluorescent Carbon Dots and Their Application to Fe^{3+} Detection in Aqueous Solutions

Shuai Ye [1], Mingming Zhang [1], Jiaqing Guo [1], Jun Song [1], Pengju Zeng [1], Junle Qu [1,2], Yue Chen [1,*] and Hao Li [1,*]

[1] Center for Biomedical Optics and Photonics (CBOP) & College of Physics and Optoelectronic Engineering, Key Lab of Optoelectronics Devices and systems of Ministry of Education/Guangdong Province, Shenzhen University, Shenzhen 518060, P. R. China
[2] National Research Nuclear University MEPhI (Moscow Engineering Physics Institute), 115409, Moscow, Russian Federation.
* Correspondence: 673252749@qq.com (Y.C.); lihao000000@163.com (H.L.)

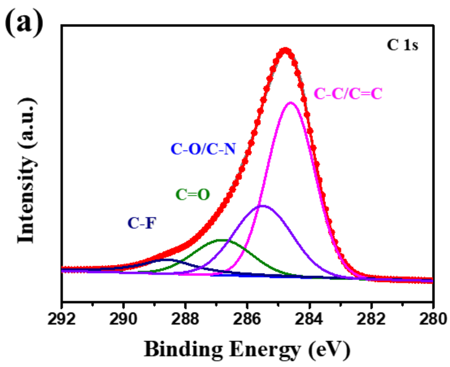

Figure S1. High-resolution (**a**) C 1s and (**b**) F 1s of XPS spectra.

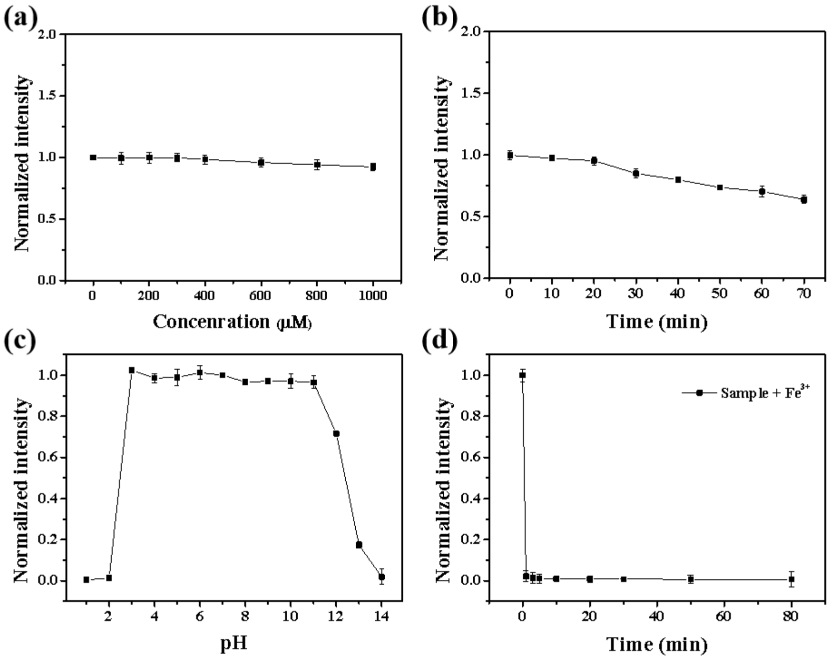

Figure S2. FNCDs fluorescence intensity at (**a**) different NaCl concentration, (**b**) different light time, (**c**) different pH and (**d**) different reaction time at 1mM Fe^{3+} concentration.

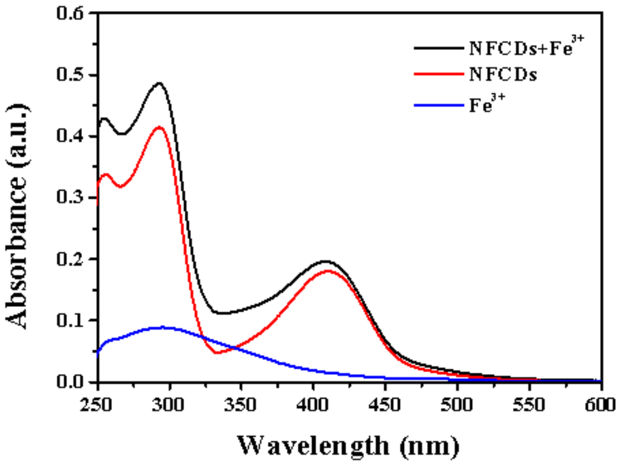

Figure S3. Absorption spectra of Fe^{3+} (blue line), NFCDs (red line), and NFCDs quenched by Fe^{3+} (black line).

Table S1. Fe^{3+} detection ability of different fluorescent CDs.

Name of Probe	The Detection Range of Fe^{3+} (μM)	The Detection Limit of Fe^{3+} (μM)	Reference
GN-CDs	0-50	0.8	[26]
CDs	33-133	0.53	[27]
CDs	0.01–2.5	0.002	[28]
CDs	0.2−1	0.073	[29]
BN-CDs	0.5–80	0.1	[30]
FNCDs	0-300	0.08	This work

Reference

1. Pang, S.; Liu, S. Dual-emission carbon dots for ratiometric detection of Fe^{3+} ions and acid phosphatase. *Anal. Chim. Acta* **2020,** *1105*, 155-161.
2. Senol, A. M.; Bozkurt, E. Facile green and one-pot synthesis of seville orange derived carbon dots as a fluorescent sensor for Fe^{3+} ions. *Microchem. J.* **2020,** *159*, 105357.
3. Desai, M.L.; Basu, H.; Saha, S.; Singhal, R.K.; Kailasa, S.K. Investigation of silicon doping into carbon dots for improved fluorescence properties for selective detection of Fe^{3+} ion. *Opt. Mater.* **2019,** *96*, 109374.
4. Wang, C.; Shi, H.; Yang, M.; Yan, Y.; Liu, E.; Ji, Z.; Fan, J. Facile synthesis of novel carbon quantum dots from biomass waste for highly sensitive detection of iron ions. *Mater. Res. Bull.* **2020,** *124*, 110730.
5. Wu, H.; Pang, L.-F.; Fu, M.-J.; Guo, X.-F.; Wang, H. Boron and nitrogen codoped carbon dots as fluorescence sensor for Fe^{3+} with improved selectivity. *J. Pharm. Biomed. Anal.* **2020,** *180*, 113052.
